# Neural Correlates of Psychotherapeutic Treatment of Post-traumatic Stress Disorder: A Systematic Literature Review

**DOI:** 10.3389/fpsyt.2017.00085

**Published:** 2017-05-19

**Authors:** Kathrin Malejko, Birgit Abler, Paul L. Plener, Joana Straub

**Affiliations:** ^1^Department of Psychiatry and Psychotherapy III, University Hospital Ulm, Ulm, Germany; ^2^Department of Child and Adolescent Psychiatry and Psychotherapy, University Hospital Ulm, Ulm, Germany

**Keywords:** post-traumatic stress disorder, functional magnetic resonance imaging, review, psychotherapeutic treatment, therapy

## Abstract

**Objectives:**

Post-traumatic stress disorder (PTSD) is a common psychiatric disease with changes in neural circuitries. Neurobiological models conceptualize the symptoms of PTSD as correlates of a dysfunctional stress reaction to traumatic events. Functional imaging studies showed an increased amygdala and a decreased prefrontal cortex response in PTSD patients. As psychotherapeutic approaches represent the gold standard for PTSD treatment, it is important to examine its underlying neurobiological correlates.

**Methods:**

Studies published until August 2016 were selected through systematic literature research in the databases PubMed, PsychInfo, and Cochrane Library’s Central Register of Controlled Trials or were identified manually by searching reference lists of selected articles. Search terms were “neural correlates” OR “fMRI” OR “SPECT,” AND “therapy” AND “PTSD.” A total of 19 articles were included in the present review whereof 15 studies compared pre-to-post-therapy signal changes, six studies related pre-treatment activity to pre-to-post-symptom improvement, and four studies compared neural correlates of responders versus non-responders. The disposed therapy forms were cognitive behavioral therapy (CBT), eye movement desensitization and reprocessing, cognitive therapy, exposure therapy, mindfulness-based intervention, brief eclectic psychotherapy, and unspecified therapy.

**Results:**

Successful psychotherapy of PTSD was repeatedly shown to be accompanied by decreased activity in the amygdala and the insula as well as increased activity in the dorsal anterior cingulate cortex (dACC) and hippocampus. Elevated dACC activity prior to treatment was related to subsequent treatment success and a positive predictor for treatment response. Elevated amygdala and insula pre-treatment activities were related to treatment failure.

**Discussion:**

Decreased activity in limbic brain regions and increased activity in frontal brain areas in PTSD patients after successful psychotherapeutic treatment might reflect regained top-down control over previously impaired bottom-up processes.

## Introduction

During lifetime, 60.7% of men and 51.2% of women experience at least one potentially traumatic event such as being taken hostage or being kidnaped, experiencing or witnessing sexual or physical assault, torture, a terrorist attack, a severe car accident, a natural disaster, war, or the unexpected death of a beloved person (1). A significant proportion of these events already occurs during young age, as 26% of male and 18% of female subjects reported of having experienced at least one traumatic event in a representative community sample (*n* = 3.021, age 14–24 years) (2). Of those experiencing potentially traumatic events, 10–40% develop psychiatric symptoms of clinical relevance (3–7) such as affective disorders, substance abuse, or post-traumatic stress disorder (PTSD). The latter goes hand in hand with significant impairments of psychological and physical health (1, 7), elevated levels of suicidality (8) as well as with a decreased level of social functioning (9).

The reason why some individuals, who have experienced a traumatic event develop a PTSD while others do not, is still not clearly understood (10). Some factors like an own or family history of psychiatric disorders before the traumatic event took place, the experience of a traumatic event early in lifetime, and/or female gender were shown to be associated with an elevated risk for developing PTSD (3, 11). Furthermore, sociodemographic and ethnologic factors play an important role, when it comes to varying prevalence rates for PTSD (7, 8, 12, 13).

Neurobiological models conceptualize the symptoms of PTSD as correlates of a dysfunctional stress reaction to traumatic events (14). This hypothesis is based on observations such as changes in the hypothalamic–pituitary–adrenal axis in PTSD patients. An abnormal downregulation of cortisol seems to result in sustained, increased levels of corticotropin-releasing hormone, which leads to a higher release of adrenocorticotropic hormone stimulating the release of glucocorticoids in turn (15). The sustained glucocorticoid exposure has been suggested to promote hippocampal atrophy as indicated by neuroimaging studies showing reduced hippocampal volumes in PTSD patients (16). Some initial hypotheses have been brought up linking neurobiological changes to altered mechanisms of extinction and hyperarousal, flashbacks, and avoidance behavior as the core symptoms of PTSD (14). For example, functional imaging studies including stressful tasks (stressful scripts, cues, and trauma reminders) showed a decreased response of the prefrontal cortex and an increased amygdala response in PTSD patients (17). Following this line of thought, it has been postulated that in patients with PTSD, stimulators of the limbic stress system prevail over inhibitors. Such an impaired top-down cognitive control over limbic areas, which is frequently associated with hypoactivation in the dlPFC might also explain flashbacks (18). In PTSD, neutral cues become associated with the traumatic event and may trigger a conditioned fear response. Failure to extinguish the conditioned fear is thought to lead to the persistence of symptoms (19). It has been suggested that the interaction between amygdala and the hippocampus, as two regions that play a critical role in the consolidation of emotionally laden memories, may contribute to the intrusive nature of trauma recollection in PTSD (20).

Diverse pharmacological as well as psychotherapeutic approaches have been suggested for the treatment of PTSD, while the latter represents the gold standard and pharmacological treatment rather constitutes an adjuvant form of symptom control. Following guidelines (21), patients should be offered a trauma-focused psychotherapy such as cognitive behavioral therapy (CBT) (22), eye movement desensitization and reprocessing (EMDR) (23), or prolonged exposure therapy (ET) (24).

CBT helps patients to question and alter dysfunctional trauma-associated cognitions. *In vivo* or *in sensu* confrontation with trauma reminders helps patients to overcome their avoidance of situations and thoughts that are no longer dangerous, which leads to habituation and a reorganization of trauma memories finally (25).

During EMDR, patients mentally focus a trauma-associated disturbing image, memory, emotion, or cognition. In comparison to CBT, confrontation is rather short and interrupted by saccadic eye movements that are initiated by the therapist (25, 26). The influence of rapid eye movements is not clearly understood yet. Mechanisms discussed are (1) focusing inner thoughts and feelings on the one hand and external stimuli (e.g., finger moves) on the other, might form an ideal attention level that is necessary for reconsolidation; (2) focusing external stimuli while reprocessing traumatic memories leads to a certain degree of distraction that prevents patients from being overwhelmed by their traumatic memories during the processing stage; (3) moving fingers might induce a certain degree of relaxation (activation of the parasympathetic system), which is then conditionally associated with traumatic memories resulting in releasing effects (25, 27, 28). Thus, besides habituation and conditioning learning processes as in CBT, modulation of attentional processing and facilitation of gaining cognitive control are suggested mechanisms of action.

Prolonged ET is a manualized (24) and effective (29, 30) psychotherapy for the treatment of PTSD. Virtual reality ET is a frequently used version of ET for treating combats, by providing realistic visual, auditory, and even tactile and olfactory cues that can be presented in a graduated manner. Habituation to anxious memories seems to be one of the key mechanisms here.

All treatment forms have in common that traumatic events or memories should no longer be avoided but confrontation on a behavioral (ET) or cognitive (CBT, EMDR) level should take place until a certain degree of fear extinction and habituation has been reached. CBT and EMDR furthermore question trauma-associated dysfunctional cognitions and replace them by more functional ones (top-down processing). Habitation and altered cognitions normally result in reduced feelings of anxiety and physiological arousal. Furthermore, confrontation with aversive stimuli and memories during therapy leads to fear extinction (31). Regarding PTSD, it was argued that extinction may not be based on an erasure of original information, but may rather reflect new learning (32).

Several neuroimaging studies attempted to examine neurobiological aspects of psychotherapeutic interventions in PTSD. The impact of trauma-focused psychotherapy on the stress system is thought to be primarily mediated by learning processes (33, 34), which is expected to be represented by changes in neurobiology. Conditional learning and extinction processes have been linked to the hippocampus and basal ganglia functioning. Biased memory for negative stimuli as in depression, but also PTSD has been suggested to result from an interaction of these regions with a hyperactive amygdala. Increased reciprocal signaling in this network may lead to sustained hyperactivity (18). Furthermore, biased processing of negative information has been linked to hypoactivity of the prefrontal cortex while biased attentional processes were also related to altered ACC activation (18). Psychotherapeutic interventions addressing these processes should, therefore, influence the activation within these areas. Accordingly, previous neuroimaging studies in adults with PTSD after psychotherapeutic intervention found decreased activity in the amygdala and an increased activity in frontal areas (35).

The assessment of neural correlates of successful psychotherapeutic treatment in adolescents with PTSD might allow for tailoring treatments to the special needs and characteristics of this population and has been suggested to have a potential for the identification of neurobiological markers that can serve as predictors for subsequent therapeutic success. We present a systematic review of the current state of the literature regarding neuroimaging and psychotherapy of PTSD.

## Materials and Methods

### Search Strategy and Inclusion Criteria

Studies published until August 2016 were selected through search in the databases PubMed, PsychInfo, and Cochrane Library’s Central Register of Controlled Trials. Additional studies were identified manually by searching reference lists of selected articles. Search terms were “neural correlates” OR “functional Magnetic Resonance Imaging (fMRI)” OR “Single Photon Emission Computed Tomography (SPECT),” AND “therapy” AND “PTSD.” The following inclusion criteria were applied: (a) published, peer-reviewed original articles, (b) studies focusing on PTSD, (c) assessment of neural correlates of PTSD by means of SPECT or fMRI, (d) active paradigms or resting state, (e) psychotherapeutic treatments, and (f) longitudinal design with respect to PTSD symptom assessment. Initial screening (titles and abstracts) and check for eligibility criteria on full-texts were conducted independently by the first author (Kathrin Malejko) and the last (Joana Straub) author. Discrepancies were finally resolved by consensus.

## Results

### Search Results and Study Characteristics

All together, 55 references were initially identified through database search and abstracts were read. One study was found by searching reference lists of selected articles manually. Finally, 19 articles were included in the present review. The other articles either did not deal with psychotherapeutic treatments, reported results of cross-sectional designs, or reported morphological data. The mean number of participants per study was *M* = 16.53 (SD = 8.04; span: 6–41), and studies were predominantly conducted in adults (*n* = 17) and only two in minors. Patients suffering from PTSD reported the experience of a vehicle accident in two studies, five studies were conducted on patients with a history of physical or sexual assault, five on war related-, two on job related- (e.g., as policeman), and five studies on various traumatic events. Ten studies also included a control group, such as PTSD waitlist patients (*n* = 2), healthy trauma controls (HTC) without PTSD (*n* = 6), healthy controls (HC) without the experience of a trauma (*n* = 3) and no control group (*n* = 8) (several answers possible). Neuroimaging assessments were done by means of fMRI (*n* = 14) and SPECT (*n* = 5). In two fMRI studies, resting state brain activation was assessed, the others applied an emotional face paradigm (*n* = 4), paradigms with other emotional content (*n* = 3), trauma script *(n* = 4), an (emotional) stroop task (*n* = 3), or a Go/NoGo task (*n* = 2). The disposed therapy forms were CBT (*n* = 9), EMDR (*n* = 4), cognitive therapy (*n* = 1), ET (*n* = 2), mindfulness-based intervention (*n* = 1), brief eclectic psychotherapy (*n* = 1), and unspecified psychotherapy (*n* = 1). Seventeen studies compared pre-to-post-therapy signal changes and two studies related pre-treatment activity to pre-to-post-symptom improvement.

### Neurobiological Markers

The majority of studies included in the present literature review found that clinical symptom improvement went along with pre-to-post-treatment signal reductions in the (left-/middle-) insula (three out of four studies) (36–39) and amygdala (four out of five studies) (35, 37, 38, 40, 41) as well as with signal enhancement in the dACC (five out of six studies) (36, 37, 39–42), hippocampus (two out of three studies) (35, 43, 44), PFC (three out of five studies) (35, 37, 40–42), and vACC (two out of three studies) (35, 43, 44). Less consistent results, regarding the direction of pre-to-post-differences, were found for the frontal cortex, thalamus, and temporal gyrus (35, 43, 45–47). Inconsistent results might be drawn back on different paradigms and control groups applied as well as to divergent types of underlying traumas.

Elevated pre-treatment activities in the insula and amygdala were associated with treatment failure (three out of four studies) (39, 48, 49). However, enhanced pre-treatment dACC activity was related to subsequent treatment success (two out of three studies) (37, 39, 48) as well as altered pre-treatment activities in the frontal cortex and IPL (16, 50, 51).

Treatment responders revealed decreased ventral insula activity and increased dACC while non-responders revealed increased amygdala and vACC activity prior to treatment (48, 51).

Taking a closer look at the insula, studies named inferior (36) and anterior areas (37) as important. Considering the ACC more closely, authors found activation of the rostral (43), subgenual (44), and dorsal (36) parts to be associated with successful treatment of PTSD. Also, when it comes to the frontal cortex, authors highlighted subregions such as the prefrontal lobe (35, 40), inferior frontal gyrus (43), dlPFC (37, 47), and ventromedial PFC (41).

In the following, differences concerning within- versus between-group analyses will be depicted: within-group analyses in PTSD patients revealed a decreased pre-to-post-treatment insula activity (37, 38) and the comparison with HTC showed heterogeneous findings (36, 39). A decreased pre-to-post-treatment amygdala activity in PTSD patients were found in both, within-group analyses (37, 38) and when being compared to PTSD waitlist patients (35, 40). Within-group analyses with respect to pre-to-post-treatment activity changes in the hippocampus revealed heterogeneous results (43, 44) and the comparison of PTSD patients with PTSD waitlist patients (35) showed a pre-to-post-signal increase in the hippocampus and vACC. In PTSD patients, increased pre-to-post-treatment dACC activity was shown for within-group analyses (37) as well as compared to PTSD waitlist patients (40, 42). There was no difference between PTSD patients and PTSD waitlist patients found before treatment (42). However, heterogeneous activity changes were shown when being compared to HTC (36, 39, 41). PTSD patients after successful treatment revealed decreased within-group activity (37) and increased pre-to-post-treatment activity in the PFC as compared to PTSD waitlist patients (35, 42). PTSD patients showed an increased global tracer uptake compared to HTC, which disappeared after treatment (50). In PTSD patients, an increased pre-treatment activity was found in the insula, dACC, and frontal gyrus compared to HTC (36, 47).

Only a few studies compared activity levels of patients with PTSD to activity levels of HC (41, 48). Bryant et al. (48) compared pre-treatment activity in PTSD patients with HC and found elevated dACC activity levels in treatment responders compared to HC. However, none of them studied whether activity levels post-treatment indeed approximate those of HC.

**Figure 1:**
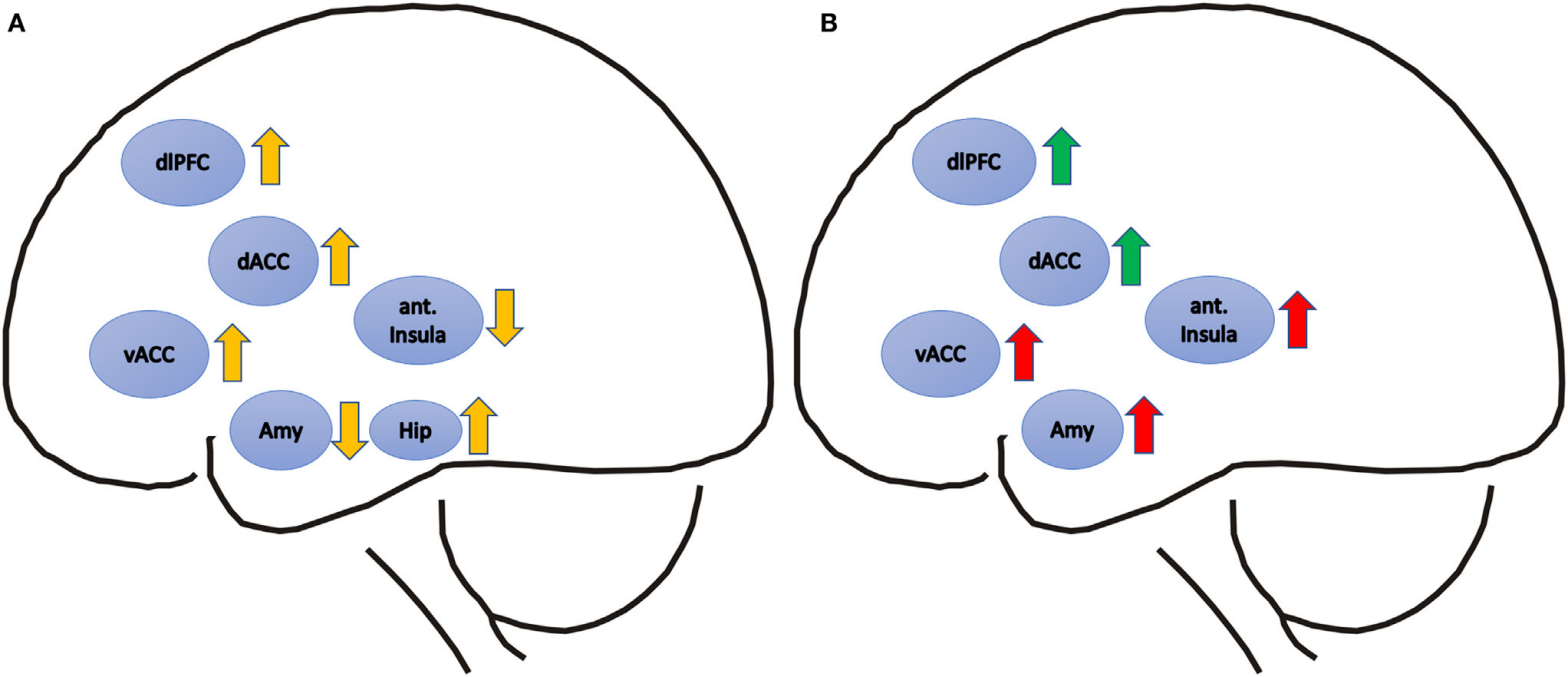
**(A)** Pre-to-post-treatment signal differences in post-traumatic stress disorder patients are depicted with yellow arrows. Upward arrows represent relatively increased, downward arrows relatively decreased activation after treatment. **(B)** Positive correlations of pre-treatment functional magnetic resonance imaging (fMRI) activity and symptom improvement are depicted with green arrows, and positive correlations of pre-treatment fMRI activity and symptom persistence are depicted with red arrows. Brain regions: dACC, dorsal anterior cingulate cortex; vACC, ventral anterior cingulate cortex; ant. Insula, anterior Insula; dlPFC, dorsolateral prefrontal cortex; Amy, amygdale; Hip, hippocampus.

For detailed results, see Table 1 and Figure 1.

**Table 1:** **Studies that investigated neural correlates of psychotherapeutic treatment of PTSD**.

Author	*N*/age group	Type of trauma	Type of control group	Psychotherapy	Paradigm, method, design	Clinical outcome	Association between pre-to-post-fMRI activity- and pre-to-post-symptom improvement	Association between pre-treatment fMRI activity and symptom improvement	Differences between responders and non-responders	Differences between PTSD patients and control group
Levin et al. (45)	6 adults	Various	None	EMDR	Trauma script, SPECT, and pre–post	– CAPS pre = 62, post = 31 – Davidson: pre = 57, post = 30 – IES: pre = 33, post = 13	– ↑ ACC, left frontal lobe			
Lansing et al. (46)	6 adults	Job related (police men)	None	EMDR	Resting state, SPECT, pre–post	– PDS: pre = 43.2, post = 5.2*	– ↓ right thalamus, right frontal lobe, left parietal lobe, occipital lobe – ↑ left frontal lobes			
Felmingham et al. (43)	8 adults	Vehicle accident; physical or sexual assault	None	CBT	Emotional face paradigm, fMRI, pre–post	– CAPS: pre = 78.1, post = 28.9	– ROI analyses: ↑ bilateral vACC – whole brain: ↑ left middle temporal gyrus, right IFL, left parieto-temporal gyrus, right hippocampus – whole brain: ↓ right post-central gyrus, right middle temporal gyrus, left superior temporal gyrus			
Pagani et al. (50)	15 adults	Job related (train drivers)	HTC (*N* = 27); R (*N* = 11) versus NR (*N* = 4)	EMDR	Trauma script, SPECT, pre–post	– SCID-I	– No regional differences found		– Pre: no significant regional differences – Post: differences in frontal, parieto-occipital-, visual cortex, hippocampus	– Pre: significant Global tracer Distribution Difference – Post: difference disappeared
Peres et al. (35)	16 adults	Various	PTSD waitlist patients (*N* = 11)	CBT	Trauma script, SPECT, pre–post	– SCID-I – CAPS: pre = 45. Post = 20* – IES: pre = 35, post = 6*	– ↑ left vACC, parietal lobes, left hippocampus, thalamus, left prefrontal cortex, bilateral thalamus – ↓ amygdala			
Bryant et al. (48)	14 adults	Various	HC (*N* = 14); R (*N* = 7) versus NR (*N* = 7)	CBT	Emotional face paradigm, fMRI, pre	– CAPS: pre = 75.5, post = 38.6		– ↑ Pre-treatment activity in bilateral amygdala and vACC in response to masked fearful faces → ↓ symptom improvement – ↑ pre-treatment activity in bilateral dACC activity → ↑ symptom improvement	– Treatment NR: ↑bilateral amygdala and right vACC activation prior to treatment than R – R: ↑ dACC prior to treatment compared to NR	– Treatment NR: ↑ bilateral amygdala and right vACC activation prior to treatment than HC – R: ↑ dACC prior to treatment compared to HC
Lindauer et al. (47)	20 adults	Various	HTC (*N* = 15); R (*N* = 10), PTSD waitlist (*N* = 10)	BEP	Trauma script, SPECT, pre–post	– SI-PTSD: pre = 11.7, post = 3.7**	– ↓ right middle frontal lobe			– Pre: ↑ activation right insula and frontal gyrus in PTSD patients versus HTC – Post: no results reported
Roy et al. (40)	8 adults	War related	PTSD waitlist patients	ET	Stroop task, fMRI, pre–post	– PCL-M: pre = 62.7, post = 51.2 – CAPS: pre = 81.8, post = 74.1	– ↓ amygdala, subcallosal gyrus, lateral PFC – ↑dACC			
Dickie et al. (44)	18 adults	Various	None	Unspecified	Emotional face paradigm, fMRI, pre–post	– CAPS: pre = 80.6, post = 44.7	– ↓ hippocampus, vACC			
Thomaes et al. (36)	16 adults	Physical or sexual assault	HTC (*N* = 22)	CT (*N* = 9) TAU (*N* = 7)	Stroop task, fMRI, pre–post	– CAPS: pre = 88.5, post = 66.2**	– ↓ dACC, left anterior insula			– Pre: ↑ anterior insula, dACC – Post: no results reported
Aupperle et al. (37)	14 adults	Physical assault	None	CBT	Other emotional paradigm, fMRI, pre–post	– CAPS: pre–post-FU**	– ↓ anterior insula, dlPFC, amygdala – ↑dACC	– Pre-treatment dACC and anterior insula activation predict treatment response		
Falconer et al. (52)	13 adults	Physical assault; vehicle accident	None	CBT	Go/noGo, fMRI, pre	– CAPS: pre = 75.5, post = 38.6		– ↑ activation pre-treatment in the left dorsal striatal and frontal networks predict treatment response		
Simmons et al. (51)	24 adults	War related	R (*N* = 9) versus NR (*N* = 15)	ET	Other emotional paradigm, fMRI, pre–post	– CAPS-R: pre = 86.7, post = 25.8 – CAPS-NR: pre = 91.1, post = 75.1			– R: ↓ ventral AI – NR: no change	
Roy et al. (41)	19 adults	War related	HTC (*N* = 18)	ET	Stroop task, fMRI, pre–post	– CAPS: pre = 80.44, post = 64.5	– ↓ amygdala – ↑ vmPFC, dACC *N* = 10			– Controls showed no significant change on repeat scanning (3–4-month interval) – No post-differences between patients and HTC reported
Cisler et al. (49)	23 minors	Physical or sexual assault	None	CBT	Emotional face paradigm, fMRI, pre	– UCLA: pre = 36.04, post = 18.30		– Pre-treatment amygdala reactivity to threat stimuli relative to neutral stimuli → ↑ improvement – ↑ amygdala activation to both threat and neutral images → ↓ symptom reduction (i.e., less threat-safety discrimination)		
van Rooij et al. (16)	41 adults	War related	HTC (N = 22); R (*N* = 22) versus NR (*N* = 17)	CBT and/or EMDR	Go/noGo, fMRI, pre–post	– CAPS-R: pre = 71.7, post = 28.1 – CAPS-NR: pre = 70.3, post = 66.1	– ↓ of IPL activity in the R-group	– Pre-treatment left IPL activation predicts treatment response	– T1: R ↑ left IPL compared to NR – T2: no difference between groups	– Less deactivation of the left motor cortex during reactive inhibition – Less activation in the rIFG during contextual cue processing
Cisler et al. (38)	20 minors	Physical or sexual assault	None	CBT	Emotional face paradigm, fMRI, pre–post	– UCLA: pre = 38.34, post – Symptom reduction slope = −0.97	– ↓ right amygdala, left middle insula FC			
King et al. (42)	12 adults	War related	PTSD waitlist patients (*N* = 8)	MBET; active control group	Resting state, fMRI, pre–post	– CAPS: pre = 72.29, post = 56.71*	– ↑ dlPFC, dACC			– Pre: no significant difference – Post: ↑ frontal cortex and ACC
Rooij et al. (39)	43 adults	war related	HTC (*N* = 25); R (*N* = 21) versus NR (*N* = 22)	CBT	Other emotional paradigm, fMRI, pre–post	– CAPS-R: pre = 66.3, post = 24.3 – CAPS-NR: pre = 74.4, post = 66.0	– ROI analyses: ↑ dACC, insula	– ↑ dACC, insula, and amygdala activation pre-treatment predict symptom persistence		– Pre and Post: ↑ insula and dACC – NR shows ↑ dACC and insula at pre and post compared to R and HTC – Amygdala/hippocampus/vmPFC: no differences at pre and post between groups

General: *pre-to-post-difference significant at p < 0.05; **p < 0.001.

*↑, pre-to-post-signal increase; ↓, pre-to-post-signal decrease; FC, functional connectivity; fMRI, functional magnetic resonance imaging; HC, healthy controls; HTC, healthy trauma controls; NR, non-responder; R, responder; T1, before treatment; T2, after treatment; TAU, treatment as usual. Brain regions: dACC, dorsal anterior cingulate cortex; dlPFC, dorsolateral prefrontal cortex; IFG, inferior frontal gyrus; IPL, inferior parietal lobe; vACC, ventral anterior cingulate cortex; vmPFC, ventromedial prefrontal cortex; PFC, prefrontal cortex. Psychotherapy forms: BEP, brief eclectic therapy; CBT, cognitive behavioral therapy; CT, cognitive therapy; EMDR, eye movement desensitization processing; ET, exposure therapy; MBET, mindfulness-based exposure therapy. Diagnostic instruments: CAPS, Clinician-Administered PTSD Scale; Davidson, Davidson Self-Rating PTSD Scale; IES, Impact of Event Scale; PCL-M, PTSD Checklist-Military Version; PDS, Foa Post-traumatic Stress Diagnostic Scale; SCID-I, Structured Clinical Interview for DSM-IV Axis I; SI-PTSD, Structured Interview for Post-traumatic Stress Disorder; UCLA PTSD Reaction Index, University of California Los Angeles PTSD Reaction Index*.

## Discussion

Results of the present systematic literature review regarding the neurobiology of PTSD treatment indicate that symptom improvement was shown to be accompanied with a pre-to-post-signal reduction in the amygdala. Accordingly, high amygdala activation prior to treatment was associated with subsequent treatment failure or non-response to treatment. Additionally, pre-to-post-signal decrease in the insula was associated with symptom improvement and increased insula activity prior to treatment was shown to be related to treatment resistance. Besides that symptom improvement goes hand in hand with fMRI signal increase in the hippocampus, dACC, PFC, and vACC, elevated dACC activity prior to treatment was shown to be related to subsequent treatment success and was a positive predictor for treatment response. Regarding cerebral networks involved, there were not obvious differences between CBT as the predominant treatment in nine of the studies listed, and ET or EMDR that were applied in three studies each. This may be interpreted in a way that changes in brain functioning rather represent changes in clinical symptoms and improvement than the different techniques that facilitate these changes. This interpretation is furthered by the finding that these therapy forms seemed to be equally effective. Therefore, changes in cerebral networks can hardly be related to distinct types of therapy but rather to elements like habituation, extinction learning, cognitive control, or attention direction.

The insular cortex is involved in emotions, salience, consciousness, motor control, and homeostasis (54, 55). Task-related fMRI studies showed increased activation in the insular cortex in individuals with PTSD, and this activation was positively correlated with the severity of PTSD symptoms (56–58). Neuroimaging studies investigating anxiety disorders also showed greater insular activation (59), pointing to a role in anxiety processing in general. With respect to the latter, the insula can be functionally divided into anterior and posterior parts: anterior insula activation, as shown to relate to salience and error processing (54), has been interpreted as a warning signal that is associated with symptoms of hyperarousal (51), whereas the posterior insular cortex is more strongly linked to the representation of interoceptive/bodily state changes, such as sympathetic arousal during negative affective states (53). Concerning the results of the present review, psychotherapeutic treatment success was associated with pre-to-post-decrease of insula activity especially in anterior parts. This can be interpreted as a correlate of reduced alertness and hyperarousal with traumatic and other emotional stimuli after treatment (55). Regarding therapeutic techniques, these changes could relate to interventions strengthening cognitive control and modulating attention as used in CBT.

Previous fMRI studies consistently showed increased amygdala activation in patients with PTSD compared to controls (60–63) and, with respect to the present review, a reduction of pre-to-post-treatment activity. As the amygdala has been suggested to play a crucial role in the processing of threatening stimuli, fear learning and fear expression (64, 65), the findings at hand might be a neurobiological correlate of a reduced response to threatening stimuli after treatment. Habituation to aversive stimuli as a result of confrontation treatment which is an element of CBT, EMDR, and ET could be a process underlying this observation. Furthermore, the present review highlights that an increased amygdala activity during fear processing prior to treatment was associated with subsequent treatment failure and non-response (48). The authors concluded that those patients, who reveal excessive fear-related amygdala activity, might have problems in regulating upcoming anxiety during confrontation with traumatic memories in therapy, which in turn might be a necessary requirement to experience subsequent habituation. In line with this, Cisler et al. (49) found that an increased amygdala reactivity to both neutral and threat stimuli before treatment was associated with poor treatment response in adolescents with PTSD. However, increased amygdala activity to threat relative to neutral stimuli was related to symptom improvement. The study of Cisler et al. (49) highlights the influence of paradigms and ways of analyses applied.

The amygdala and the hippocampus have both been suggested to play critical roles in the consolidation of emotionally laden memories in healthy adults. The interaction between these two hyper-responsive regions may contribute to the intrusive nature of trauma recollection in PTSD (20). Prolonged exposure to stress such as in patients with PTSD induces high glucocorticoid levels, which seem to harm the hippocampus (impairment of neurogenesis and loss of dendritic spines) (66). Structural imaging studies showed that PTSD patients have reduced hippocampal volume compared to HC (67–70). Whether the smaller hippocampal volumes in PTSD patients are associated with functional abnormalities still remains unclear. fMRI studies that compared PTSD patients with HC revealed both increased (71) and decreased hippocampal activation (72). The present review found pre-to-post-treatment increase of the hippocampus activity. It has been speculated that this might relate to improved short- and long-term consolidation and memory processes, improved encoding and recognition of episodic memory as well as an improved evaluation of experiences (35, 66). Furthermore, increased hippocampus activity might point toward improved identification of safe contexts, improved learning, and alteration of existing memories (20). Habituation and exposition as integral parts of the majority of treatments applied may be linked to these changes observed.

In the context of the processing of traumatic events, the ACC has been implicated in helping modulate fight/flight reactions to perceived threats (45). Altered activity in the ACC after successful psychotherapy of PTSD was, therefore, interpreted as a correlate of improved conflict monitoring and emotion regulation (37). The ACC can be subdivided functionally in ventral and dorsal parts: the vACC has been proposed to play a particular role in emotion processing, automatic emotion control, and social decision making (37, 73). The vACC was shown to be interconnected with the amygdala, nucleus accumbens, hypothalamus, and anterior insula and is assumed to be involved in assessing the salience of emotion and motivational information (74). The dACC has been proposed to be involved in cognitive processing or appraisal of emotion and the saliency of the source of social information (37, 73). The dACC is particularly connected with the prefrontal cortex and parietal cortex as well as the motor system and the frontal eye fields, making it a central node for processing top-down and bottom-up stimuli and assigning appropriate control over other areas in the brain (74). Following the results of the present review, pre-to-post-enhancement of dorsal and ventral ACC activity might be a correlate of improved cognitive processing of trauma-associated cognitions, better appraisal of trauma-associated emotions as well as in improved emotion control, all of these being processes that are directly addressed particularly in CBT. The present review found that treatment success was rather associated with an elevated pre-treatment activity in the dACC, which in turn might relate to better preconditions regarding cognitive control and emotion regulation.

Frontal brain areas have been strongly associated with PTSD in the literature before. The majority of studies revealed an increased pre-to-post-treatment activity in the PFC, which might be interpreted as a correlate of improved top-down regulation of emotional systems as well as a potential correlate of improved processing of risk and fear, decision making and emotion regulation (20, 75) as established during psychotherapy, particularly CBT. Changed prefrontal functioning might also relate to findings from structural neuroimaging studies that found reduced volumes in prefrontal brain regions (75–77) in PTSD patients. In a similar vein, results of a connectivity study of King et al. (42) showed increased connectivity to dlPFC regions within the central executive network after successful PTSD treatment, which was interpreted as a potential correlate of improved emotion regulation. The dlPFC was shown to play a critical role in working memory. Regarding PTSD, it was argued that extinction may not be based on an erasure of original information, but may rather reflect new learning (32). Increased activation in the dlPFC in the context of successful psychotherapy might represent a correlate of newly gained control of unwanted memories (47).

Post-traumatic stress disorder patients after successful treatment point toward a predominantly increased pre-to-post-treatment activity in “cognitive brain areas” like the PFC, dACC, and hippocampus when being compared to PTSD waitlist patients (35, 40, 42). Decreased pre-to-post-treatment activity in “emotional brain areas” like the insula and amygdala was more pronounced in within-group analyses (37, 53) and compared to patients of the waitlist control group (35, 40). Those findings underscore the importance of taking into consideration the control group applied when interpreting data of treatment studies.

Among others the impact of the review presented here might, therefore, be limited due to cross-study variations in control groups applied, different trauma histories, subject characteristics, and paradigms applied as this makes it very difficult to draw valid conclusions regarding subgroup and overall results.

Neurocircuitry models emphasize reduced activation of frontal brain areas and elevated activity of limbic regions in patients with PTSD and a loss of top-down inhibition is thought to be one of the main factors resulting in impaired extinction of traumatic memories or insufficient modulation of affect (e.g., re-experiencing) (20). The present review was able to show that brain activation results partly fit into this picture with relatively consistent signal increases in the dACC as a frontal brain area, while activity in key areas related to emotional processing like the anterior insula and amygdala rather decreased from pre-to-post-psychotherapy. This can be seen as an improved bottom-up and top-down regulation in response to successful treatment. Future studies should investigate whether abnormal activity patterns in patients with PTSD indeed normalize after treatment and become comparable to those of HC.

## Author Contributions

JS and KM contributed substantially to the work, analyzed and interpreted the data, and drafted the manuscript. BA and PP contributed substantially to the work, interpreted the data, and revised the manuscript critically for important intellectual content. All the authors approved the final version to be published and agreed to be accountable for all aspects of the work in ensuring that questions related to the accuracy or integrity of any part of the work are appropriately investigated and resolved.

## Conflict of Interest Statement

All authors have completed the ICMJE uniform disclosure form at www.icmje.org/coi_disclosure.pdf and declare: no support from any organization for the submitted work and no other relationships or activities that could appear to have influenced the submitted work.
